# Real-world use of angiotensin-converting enzyme inhibitors/angiotensin receptor blockers/β-blocks in Chinese patients before acute myocardial infarction occurs: patient characteristics and hospital follow-up

**DOI:** 10.1186/s12967-018-1720-3

**Published:** 2018-12-10

**Authors:** Xuhe Gong, Xiaosong Ding, Hui Chen, Hongwei Li

**Affiliations:** 1 Department of Cardiology, Cardiovascular Center, Beijing Friendship Hospital, Capital Medical University, Beijing, 100050 People’s Republic of China; 2 Department of Internal Medicine, Medical Health Center, Beijing Friendship Hospital, Capital Medical University, Beijing, 100050 People’s Republic of China; 3 Beijing Key Laboratory of Metabolic Disorder Related Cardiovascular Disease, Beijing, 100069 People’s Republic of China

**Keywords:** Angiotensin-converting enzyme inhibitors, Angiotensin-receptor blockers, β-Blockers, Myocardial infarction, Mortality, Major adverse cardiovascular events (MACE)

## Abstract

**Background:**

Current guidelines recommend angiotensin-converting-enzyme inhibitors (ACEI) or angiotensin-receptor blockers (ARB) or β-blockers (β-B) for secondary prevention in patients after an acute myocardial infarction (AMI). However, there is limited data to evaluate ACEI/ARB/β-B (AAβ) used before AMI on major adverse cardiovascular events (MACE), in China patients.

**Objectives:**

This study sought to investigate whether AAβ treatment prior to AMI is associated with better hospital outcomes at the onset of AMI.

**Methods:**

A total of 2705 patients were selected from the Cardiovascular Center Beijing Friendship Hospital Database Bank, and divided into two groups on the basis of admission prescription: AAβ (n = 872) or no-AAβ (n = 1833). The study was also designed using propensity-score matching (226 AAβ treated patients vs 452 no-AAβ treated patients). The primary outcome was a composite of cardiac death and heart function and infarct size during hospitalization follow-up.

**Results:**

The mean follow-up period was about 8 days in MACE. The Cox model showed the two groups had similar risk of cardiac death. The in-hospital mortality was 3.36% (3.33% of AAβ users and 3.38% of nonusers, p = 0.94). In adjusted analysis, there was still no difference in in-hospital mortality between the two groups (3.54% vs 2.88%, p = 0.64). However, the AAβ treated patients were associated with better heart function and smaller infarct size than the no-AAβ treated patients.

**Conclusions:**

The in-hospital MACE was similar between AAβ treated patients and no-AAβ treated patients. However, treatment with AAβ before AMI was associated with improved heart function and smaller infarct size.

## Background

Ischemic heart disease is one of the most frequent diseases worldwide; and cardiovascular diseases are among the leading causes of death in developed industrial countries. With the development of China’s economy, the number of patients with acute myocardial infarction (AMI) increases year by year in China, and the overall mortality rate is on the rise [1]. Although interventional therapy has greatly improved the prognosis of myocardial infarction, the basic drug therapy is also essential. A large number of clinical trials have found that angiotensin-converting enzyme inhibitors (ACEIs), Angiotensin-receptor blockers (ARBs) and β-blockers (β-B) prevented ischemic events and mortality in patients with AMI [2–4]. Thus, secondary prevention protocols including these agents are regarded to be standard therapy following an AMI, along with aspirin and statins [5, 6]. Although there is no doubt that ACEI/ARB/β-blocker (AAβ) offer the most benefit to AMI patients, there is still uncertainty about prescribing these agents to a real population of patients before AMI occurs.

Moreover, several previous studies have documented less benefit with these agents in patients with lower-risk myocardial infarctions [7, 8]. Was it possible to improve the prognosis by taking so many drugs before myocardial infarction occurs? This is a question. In addition, most studies have a longer follow-up time, and we only focus on events during hospitalization. Therefore, by using Cardiovascular Center Beijing Friendship Hospital Database Bank, we sought to evaluate the effectiveness of AAβ treatment in improving hospital survival. This study focused on the left ventricular functions evaluated by echocardiography, myocardial infarct size estimated by peak concentration of myocardial enzyme and the major cardiovascular events (MACE) in hospital, the MACE includes cardiac-death, target vascular reconstruction, recurrent myocardial infarction, malignant arrhythmia, cerebral infarction and cerebral hemorrhage.

## Methods

### Study population

The present study was based on the Cardiovascular Center Beijing Friendship Hospital Database Bank (CBD Bank). Briefly, this is a single center study. From January 2013 to October 2016, a total of 2712 consecutive patients with AMI were enrolled in this study. The local institutional review board at our hospital approved the study protocol, and this study was in accord with the Declaration of Helsinki.

### Inclusion and exclusion criteria

Inclusion criteria for the present analysis were as follows: (1) consecutive patients 18 years of age or older; (2) patients diagnosed with ST-segment elevation AMI (STEMI) or non-ST-segment elevation AMI (NSTEMI). Exclusion criteria were (1) a lack of documentation of prescribed medications on admission; (2) both ARB and ACEI received; (3) infectious diseases (tuberculosis, active infective endocarditis), rheumatic disease (systemic lupus erythematosus, rheumatoid arthritis, vasculitis), hematological diseases (leukemia, lymphoma, disseminated intravascular coagulation) and neoplastic disease.

**Fig. 1:**
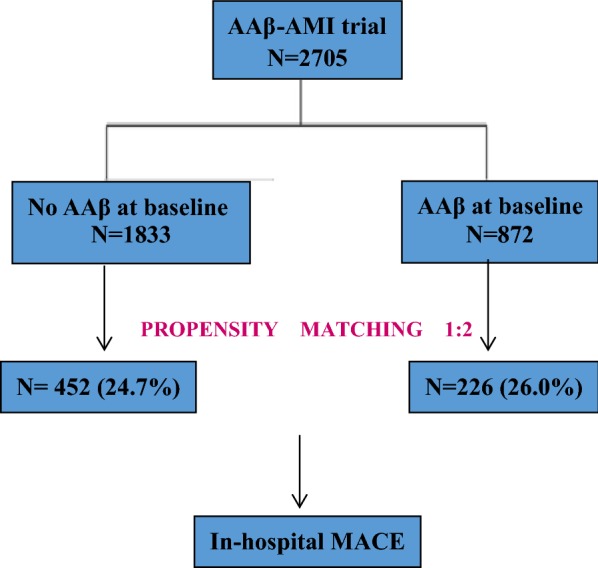
Flow chart of patient enrollment. *AAβ* angiotensin-converting-enzyme inhibitors (ACEI) or angiotensin-receptor blockers (ARB) or β-blockers (β-B), *AMI* acute myocardial infarction, *MACE* major adverse cardiovascular events

Finally, a total of 2705 patients were included in this study, the study was also designed using propensity-score matching to assemble a balanced cohort. The patient flow of the study is shown in Fig. 1.

### The basic characteristics data

The hospital medical records were detailed and intact. Most of the data was extracted from the medical records including demographic data (age and sex), history of past illness (hypertension, coronary disease, diabetes, hyperlipemia and other diseases), conditions of smoking and drinking, family histories [hypertension, diabetes and coronary heart disease (CHD)] and medications (ACEI, ARB, β-blocker and other) before admission. Body mass index (BMI) was calculated by dividing weight in kilograms by height in meters squared (kg/m^2^).

We analyzed baseline demographic characteristics, history of past illness, initial laboratory test results and medications. Blood samples for baseline laboratory tests were collected at admission or during the first 5 days after presentation of acute myocardial infarction. Serum peak concentration of cardiac troponin I (cTnI), myoglobin (Myo), creatine kinase-myocardial band (CKMB) level were used for estimation of infarct size. The LV ejection fraction was determined using 2-dimensional echocardiography during the index hospitalization. In-hospital complications and their management were also recorded.

The major adverse cardiac events (MACEs) in hospital were defined as cardiac death, target vascular reconstruction, malignant arrhythmia, recurrent myocardial infarction, cerebral infarction and cerebral hemorrhage.

### Data analysis

Continuous variables are presented as mean ± standard deviations or median with interquartile range, and were compared using the unpaired Student’s t tests or the Mann–Whitney U test. Categorical variables are expressed as frequencies and percentages, and were compared by Chisquare or Fisher’s extract statistics. Patients were categorized into two groups: patients receiving AAβ, and patients no receiving AAβ. Since patients were not randomly assigned to AAβ or no-AAβ, 1:2 propensity score (PS) matching based on their probability of using AAβ was performed to reduce the effect of treatment-selection bias and potential confounding factors in this observational study. For each patient, a PS indicating the likelihood of using AAβ before hospitalization was calculated using a non-parsimonious multivariable logistic regression model with covariates including baseline demographic characteristics, such as age, sex and body mass index (BMI), past medical history including hypertension, diabetes mellitus, hyperlipidemia, heart failure, renal dysfunction and coronary heart disease.

Survival curves were conducted using Kaplan–Meier estimates and compared with the log-rank test. The multivariate Cox proportional hazards regression analysis was used to assess the association between adverse clinical events and the AAβ and no-AAβ groups. All factors showing significance in the univariate analysis (p < 0.05) were then examined by a multivariate analysis.

All statistical tests were two-tailed, with statistical significance defined as a p value of < 0.05. All analyses were performed by using SAS 9.4 (SAS Institute Inc., Cary, NC, USA) and Metaninf function in Stata 12.0.

## Results

### Baseline characteristics

#### Overall population

Among the 2705 eligible patients, AAβ were prescribed to 872 patients (32.2%), and no-AAβ group was 1833 patients (67.8%) pre-admission. The median age was 65 years (interquartile range 56–77 years); 70.9% of the patients were men. A total of 33.9% of the patients had diabetes, 65.4% had hypertension, and just 1.29% of patients had LV systolic dysfunction. The baseline clinical characteristics are shown in Table 1.

**Table 1:** Baseline characteristics

Characteristics	Before PS match		*p* value	After PS match		*p* value
	AAβ (n = 872)	NO-AAβ (n = 1833)		AAβ (n = 226)	NO-AAβ (n = 452)	
Demographic						
Age (years)	68 (58–78)	63 (55–76)	< 0.001	64 (55–77)	66 (55–78)	0.56
Male sex	582 (66.7)	1336 (72.9)	0.001	155 (68.6)	319 (70.6)	0.60
BMI (kg/m^2^)	25.6 (23.4–27.9)	25.0 (22.9–27.4)	0.001	25.4 (23.4–28.0)	25.3 (22.9–27.6)	0.28
Initial presentation						
SBP (mmHg)	133 (122–149)	126 (112–140)	< 0.001	130 (117–144)	130 (118–144)	0.93
DBP (mmHg)	74 (67–82)	72 (65–80)	< 0.001	74 (67–83)	74 (65–82)	0.50
Killip class ≥ 2	318 (36.5)	531 (29)	< 0.001	65 (28.8)	130 (28.8)	0.23
Past history						
HT	836 (95.9)	940 (51.4)	< 0.001	191 (84.5)	382 (84.5)	1
DM	376 (43.1)	550 (30.0)	< 0.001	29 (12.8)	67 (14.8)	0.48
Dyslipidemia	414 (47.6)	689 (38.2)	< 0.001	78 (34.5)	146 (32.3)	0.56
Smoking	460 (52.8)	1144 (62.4)	< 0.001	122 (54.0)	262 (58.2)	0.29
CRF	96 (11)	101 (5.5)	< 0.001	14 (6.19)	23 (5.09)	0.55
HF	24 (2.78)	11 (0.61)	< 0.001	2 (0.88)	2 (0.44)	0.48
CAD	459 (52.7)	594 (32.7)	< 0.001	35 (15.5)	71 (15.7)	0.94
Previous MI	149 (17.1)	172 (9.5)	< 0.001	22 (9.7)	35 (7.8)	0.38
Previous PCI	219 (25.1)	194 (10.6)	< 0.001	18 (8.0)	17 (3.8)	0.02
Stroke	188 (21.6)	285 (15.6)	< 0.001	41 (18.1)	81 (17.9)	0.94
Laboratory finding						
TC (mmol/L)	4.10 (3.44–4.78)	4.42 (3.77–5.08)	< 0.001	4.27 (3.61–4.86)	4.42 (3.84–5.04)	0.01
TG (mmol/L)	1.35 (0.99–1.97)	1.40 (1.0–1.97)	0.287	1.27 (0.99–1.87)	1.38 (0.99–1.96)	0.21
LDL-C (mmol/L)	2.33 (1.83–2.80)	2.55 (2.07–3.06)	< 0.001	2.41 (1.96–2.93)	2.60 (2.13–3.01)	0.004
pNT-proBNP (ng/L)	2035 (587–7645)	1637 (557–5475)	0.01	1712 (674–5693)	1701 (587–5598)	0.91
Scr (μmol/L)	88.3 (75.0–106.8)	83.5 (74.0–96.0)	< 0.001	84.2 (74.4–101.6)	84.5 (75.0–95.7)	0.51
GFR (mL/min	72.2 (55.2–87.3)	80.5 (64.1–95.1)	< 0.001	74.7 (59.8–89.3)	78.1 (63.6–93.9)	0.14
Hospital course						
STEMI	326 (37.4)	980 (53.5)	< 0.001	113 (50)	258 (57.1)	0.08
NSTEMI	546 (62.6)	853 (46.5)	< 0.001	113 (50)	194 (42.9)	0.08
E-PCI	191 (29.7)	534 (36.5)	0.003	53 (29.0)	139 (37.6)	0.046

*AAβ* ACEI/ARB/β-B, *BMI* body mass index, *SBP* systolic blood pressure, *DBP* diastolic blood pressure, *HT* hypertension, *DM* diabetes mellitus, *CRF* chronic renal failure, *HF* heart failure, *CAD* coronary artery disease, *MI* myocardial infarction, *PCI* percutaneous coronary intervention, *TC* total cholesterol, *TG* triglyceride, *HDL-c* high-density lipoprotein cholesterol, *LDL-c* low-density lipoprotein cholesterol, *NT-proBNP* N-terminal pro-brain natriuretic peptide, *GFR* glomerular filtration rate, *STEMI* ST-segment elevation AMI, *NSTEMI* non-ST-segment elevation AMI, *E-PCI* emergency percutaneous coronary intervention

*p* values for comparisons between the two groups. Significance level was 0.05

**Fig. 2:**
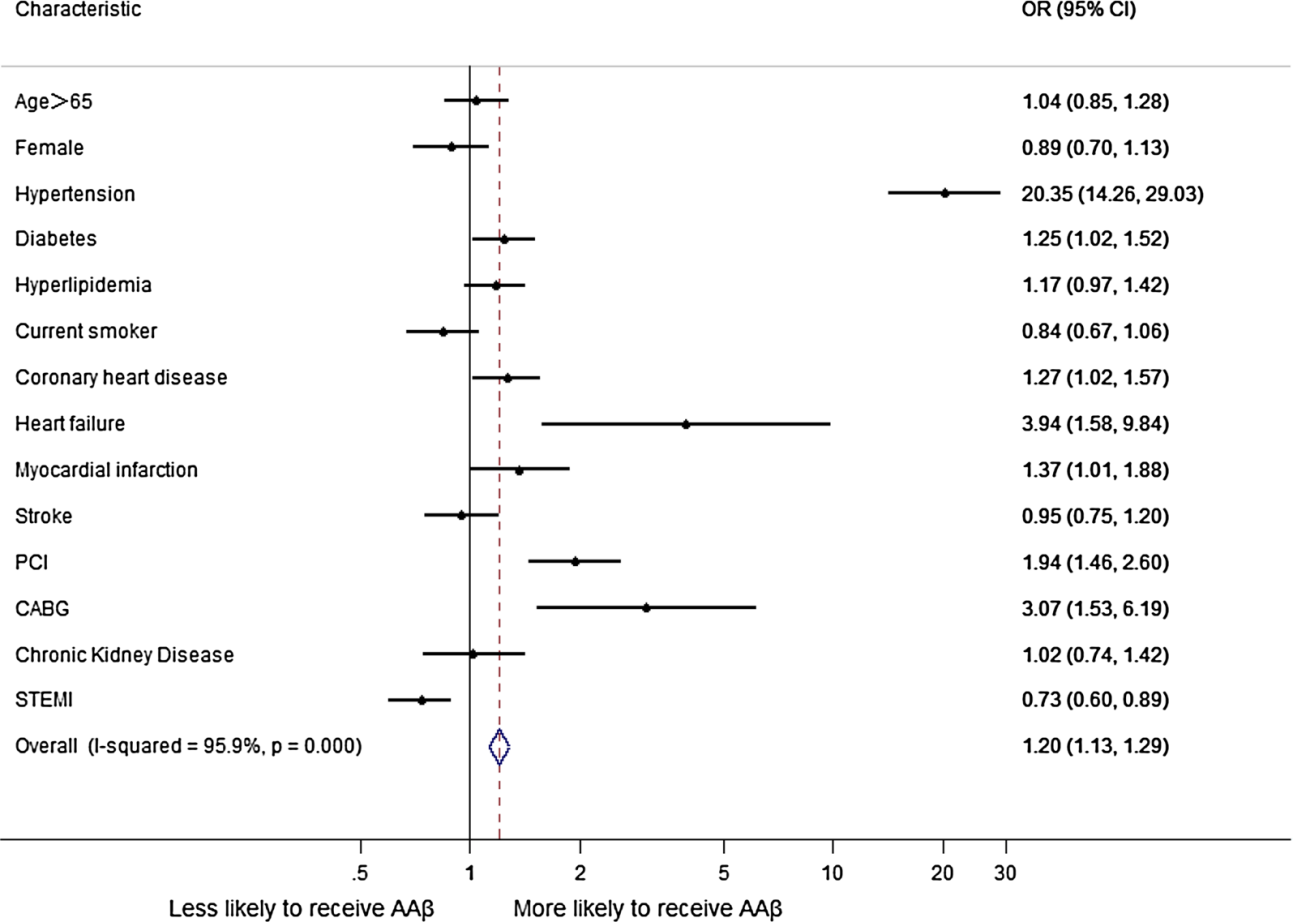
Factors associated with AAβ use in multivariable analysis. Variables associated with AAβ use are shown along the vertical axis. The strength of effect is shown along the horizontal axis with the vertical line demarcating an odds ratio (OR) of 1 (i.e., no association); estimates to the right (i.e., > 1) are associated with a greater likelihood of AAβ use, whereas those to the left (i.e., < 1) indicate a reduced likelihood of AAβ use. Each dot represents the point estimate of the effect of that variable in the model, whereas the line shows the 95% confidence interval (CI)

Significant correlates of AAβ therapy in multivariable analysis are shown in Fig. 2. Compared with no AAβ-treated patients, patients prescribed AAβ prior to admission were more likely to be women, and had worse baseline clinical: the higher BMI, the higher systolic and diastolic blood pressure at admission; what’s more, the proportion of hypertension, diabetes, heart failure, stroke, old myocardial infarction and renal insufficiency is higher. Also, the no AAβ-treated patients were more likely to have acute ST segment elevation myocardial infarction (STEMI); hence the proportion of emergency PCI (percutaneous coronary intervention) is higher.

#### Propensity score-matched population

Propensity scores for AAβ use, calculated for 678 patients, were used to match 226 patients receiving AAβ (33.3%) with 452 patients no receiving AAβ (66.7%). There were no significant differences in baseline clinical, past medical history, types of myocardial infarction between the AAβ treated and no- AAβ treated patients for the propensity score-matched subjects, except for previous PCI (p = 0.02, Table 1).

The estimated infarction size and left ventricular function between AAβ treated patients and no-AAβ treated patients.

Serum peak concentration of cTnI, Myo and CKMB level were used for estimation of infarct size. We found no difference in pMyo between the two groups, however, there was higher peak level of serum myocardial enzymes (p-CKMB and p-cTnI) in the no-AAβ treated patients. (p-CKMB: 57.0 vs 31.9, p = 0.02, p-cTnI: 6.0 vs 3.0, p = 0.002, Table 2).

**Table 2:** The estimated infarction size between AAβ and NO-AAβ group

The peak value of myocardial enzyme	Before PS match		*p* value	After PS match		*p* value
	AAβ (n = 872)	NO-AAβ (n = 1833)		AAβ (n = 226)	NO-AAβ (n = 452)	
pMyo (U/L)	69.4 (33–172)	75 (32.8–228)	0.12	75.2 (30.1–184.5)	75.9 (34.8–224.5)	0.32
pCK-MB (ng/mL)	28.3 (6–105)	47.9 (8.8–164)	< 0.001	31.9 (8.7–111)	57 (10–164)	0.02
pcTnI (ng/mL)	3.3 (0.64–11.0)	5.1 (1.2–17.1)	< 0.001	3.0 (0.72–10.0)	6.0 (1.4–21.1)	0.002

*AAβ* ACEI/ARB/β-B, *Myo* myoglobin, *CK-MB* creatine kinase-myocardial band, *cTnI* cardiac troponin I, *p* peak value of

*p* values for comparisons between the two groups. Significance level was 0.05

From the perspective of cardiac function assessed by echocardiography, the AAβ treated patients were associated with better heart function and smaller infarct size than the no-AAβ treated patients. In terms of cardiac function evaluation, the left ventricular ejection fraction (EF) and fraction shortening (FS) in the AAβ treated patients were significantly higher than the no-AAβ treated patients. (EF: 0.63 vs 0.61, p = 0.009, FS: 0.34 vs 0.33, p = 0.004, Table 3).

**Table 3:** The comparison of left ventricular function between AAβ and no-AAβ group

Characteristic	Before PS match		*p* value	After PS match		*p* value
	AAβ (n = 872)	NO-AAβ (n = 1833)		AAβ (n = 226)	NO-AAβ (n = 452)	
LA	3.9 (3.5–4.2)	3.7 (3.4–4.1)	< 0.001	3.8 (3.5–4.1)	3.7 (3.5–4.1)	0.88
LVEDD	5.2 (4.9–5.6)	5.2 (4.8–5.6)	0.277	5.2 (4.8–5.5)	5.2 (4.8–5.6)	0.20
EF	0.61 (0.53–0.67)	0.61 (0.52–0.66)	0.029	0.63 (0.55–0.67)	0.61 (0.53–0.66)	0.009
FS	0.33 (0.27–0.37)	0.33 (0.27–0.36)	0.016	0.34 (0.29–0.38)	0.33 (0.27–0.37)	0.004
E/A	0.82 (0.69–1.17)	0.87 (0.72–1.24)	< 0.001	0.88 (0.70–1.20)	0.84 (0.70–1.19)	0.37

*AAβ* ACEI/ARB/β-B, *BMI* body mass index, *LA* left atrium, *LVEDD* left ventricular end-diastolic dimension, *EF* left ventricular ejection fraction, *FS* fraction shortening, *E/A* ratio of early to late ventricular filling velocities

*p* values for comparisons between the two groups. Significance level was 0.05

### Clinical outcomes

The median follow-up duration was 8 days (interquartile range 6–10). All-cause death occurred in 91 patients (3.4%) in the overall population. There were no significant associations between the treatment strategy and all-cause death or cardiac death. The multivariate Cox proportional hazards regression analysis showed both the AAβ treated patients and no-AAβ treated patients had similar risk of cardiac death or all-cause death (cardiac death, p = 0.72, all-cause death, p = 0.94, Table 4).

**Table 4:** Comparison of clinical outcomes during hospitalization between study groups

	AAβ (n, %)	NO-AAβ (n, %)	*p* value
Overall population			
Number	872	1833	
MACE	69 (7.92)	108 (5.92)	0.049
Cardiac-death	28 (3.21)	54 (2.96)	0.720
All-cause death	29 (3.3)	62 (3.4)	0.939
Target vascular reconstruction	1 (0.11)	0 (0)	0.148
Recurrent myocardial infarction	39 (4.48)	45 (2.47)	0.005
Malignant arrhythmia	2 (0.23)	2 (0.11)	0.451
Cerebral infarction	3 (0.35)	7 (0.39)	0.874
Cerebral hemorrhage	3 (0.35)	8 (0.44)	0.718
Matched population			
Number	226	452	
MACE	13 (5.75)	21 (4.65)	0.53
Cardiac-death	8 (3.54)	12 (2.65)	0.631
All-cause death	8 (3.54)	13 (2.88)	0.638
Target vascular reconstruction	0 (0)	0 (0)	
Recurrent myocardial infarction	6 (2.65)	7 (1.55)	0.322
Malignant arrhythmia	1 (0.44)	2 (0.44)	0.999
Cerebral infarction	0 (0)	3 (0.67)	0.220
Cerebral hemorrhage	0 (0)	4 (0.89)	0.157

MACE = cardiac-death or target vascular reconstruction or recurrent myocardial infarction or malignant arrhythmia or cerebral infarction or cerebral hemorrhage

*MACE* major adverse cardiovascular events

After propensity-score matching, all-cause death occurred in 3.5 and 2.9% of matched patients receiving AAβ and no-AAβ, no significant differences were also observed in the incidence of all-cause death between the two groups.

### Survival

**Fig. 3:**
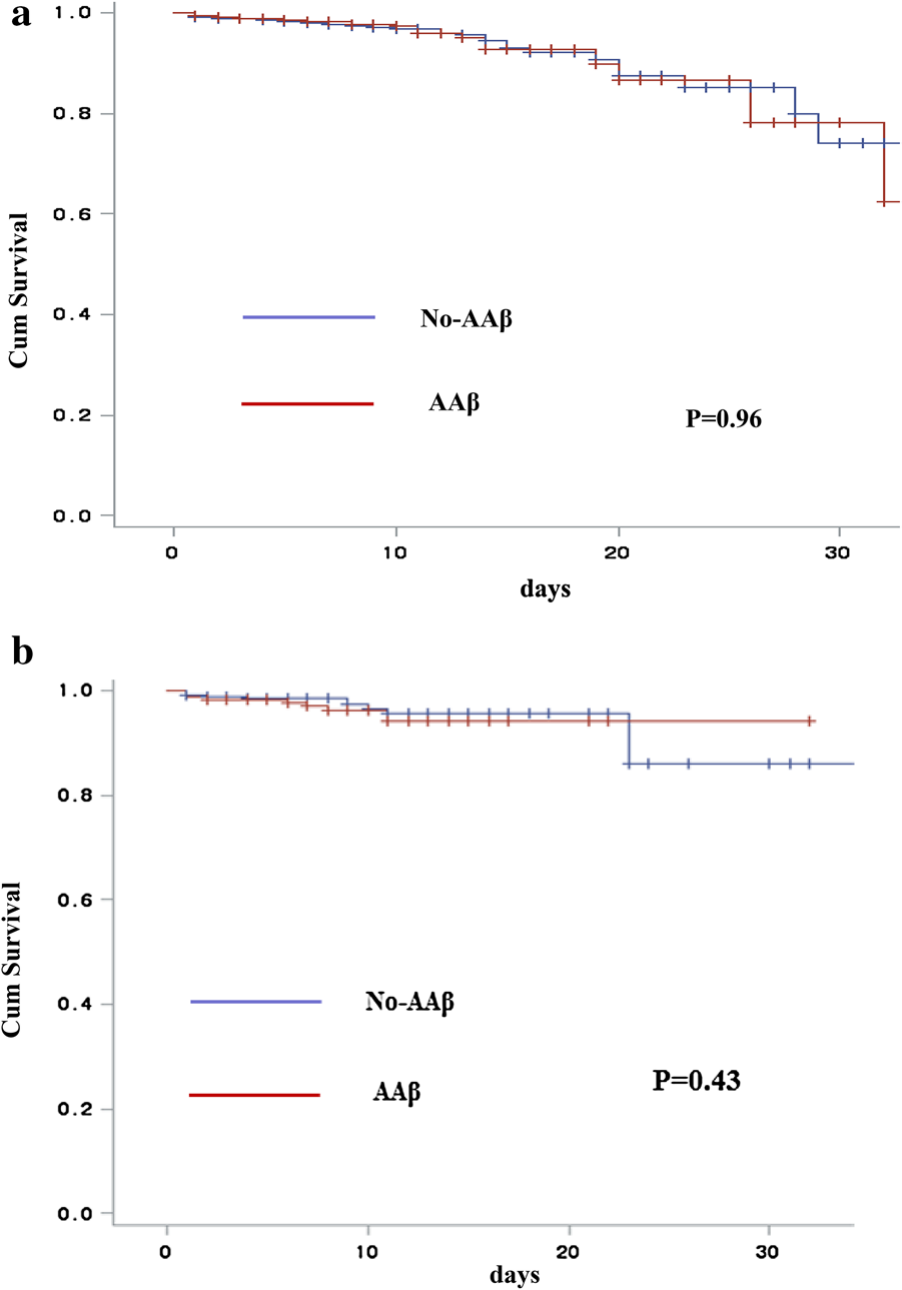
Survival curves of all-cause death during hospitalization between the groups. **a** Overall population. **b** Propensity score-matched population

In survival analysis, in-hospital death was no significant differences between the two groups. After adjusting for baseline clinical and propensity score, there were also no significant differences (Fig. 3).

## Discussion

In this single center observational study, we found that previous treatment with AAβ was associated with a non-significant reduction in the risk of all-cause mortality during hospitalization. However, previous use of AAβ reduced myocardial infarction size and improved heart function.

The clinical benefit of AAβ in patients after AMI may be partly mediated by a reduction in the risk of recurrent ischemic events and reduction in congestive heart failure [9, 10], some studies emphasizing that the AAβ have an additive effect, which have long been reflected in the clinical guidelines, which recommend routine use of AAβ in all AMI patients [11, 12]. However, Could the drugs used in the past improve the condition of myocardial infarction? Moreover, it is controversial whether this medical prevention improves clinical outcomes in hospital. Therefore, the adherence to these guideline-based medications differs substantially among cardiovascular societies [13, 14]. Thus, considering the potential adverse events attributed to over-use of AAβ treatment prior to AMI, treatment of the unselected population with these agents might be inappropriate in the modern PCI era [15]. Our current study, based on reliable data that included all AMI patient from 2013 to 2016, may provide an important ‘‘real world’’ insight into this debatable issue.

In our study population, 11.9% of patients had a history of myocardial infarction; we found that 53.6% of patients with old myocardial infarction were not taking the AAβ, which suggests that application of medicine for improving prognosis of myocardial infarction was still inadequate. A recent clinical research conducted by Liu et al. [16] that focused on the use and trends of ACEI/ARB therapy in China over the past decade (2001–2011), after analyzing 102,003 patients, they found that one-third of Chinese AMI patients with Class I indications do not receive ACEI/ARB therapy during hospitalization, with little improvement in rates over time. The underutilization of ACEI/ARB therapy was also observed in our study. Moreover, the main characteristics of patients who are more willing to take AAβ are as follows: the history of hypertension, coronary heart disease and heart failure.

In our present analysis, the all cause death was no significant difference in patients who were treated with either AAβ or not before AMI occurs. After matching, there was also no difference in mortality between the two groups. This finding may be partly explained by the short follow-up time, we only observed the deaths in the hospital. If we extend the follow-up period, for example, such as 1 year, 2 years, or longer, there may be a benefit of AAβ treatment.

Moreover, could the AAβ improve the MACE? This research found that the treatment strategy was not related to target vascular reconstruction, malignant arrhythmia, cerebral infarction and cerebral hemorrhage. The only difference was the proportion of recurrent myocardial infarction, which was reduced in the no-AAβ treated patients (2.47% vs 4.48%, p = 0.005); However, this difference disappears after matching. Therefore, the AAβ did not improve the in-hospital MACE. Similarly, as the follow-up time increases, the role of AAβ therapy in improving MACE may be apparent.

Although the hospital mortality was no difference between the two groups, the use of AAβ really reduced the size of the infarct area and improved heart function; In other words, the drugs used in the past improved the condition of myocardial infarction. The serum peak concentration of Myo, cTnI, CKMB level was used for estimation of infarct size [17]. We found no difference in the peak value of Myo between the two groups of patients, the peak value of CK-MB and cTnI were reduced in the AAβ treated patients. We analyzed that the reasons for no difference in the peak value of Myo between the two groups are as follows: first, Myo has no myocardial specificity, and it is rapidly released into the bloodstream during myocardial infarction, with high sensitivity but poor specificity. Second, Myo increased after 1–4 h of myocardial infarction and reached peak value in 6–7 h; however, some patients see a doctor after 6–7 h of myocardial infarction. The detected peak value of Myo is not the true peak value of Myo during the evolution of myocardial infarction. In any case, the peak value of CK-MB and cTnI were sufficient to represent the myocardial infarct size. The size of the infarct area was indeed reduced in the AAβ treated patients.

In terms of the type of myocardial infarction, the pre-match analysis showed that the proportion of STEMI in the no-AAβ treated patients was higher; therefore, it has more emergency PCI proportion. This finding may be partly explained by the characteristics of the AAβ treated patients because they had a more serious medical history, such as heart failure, stroke and coronary heart disease. As we are known, STEMI was transmural infarction with complete occlusion of the coronary arteries from a pathological point of view, this condition was often worse [18]. The use of AAβ reduces the incidence of STEMI. However, after adjusting for possible confounding variables, the benefits of AAβ disappeared, there was no difference in the type of myocardial infarction between the two groups.

## Limitations

Our present study had limitations inherent to its nonrandomized, observational design. First, similar to previous studies using an administrative database; we did not have full information on the dose and duration of AAβ use. Second, because china population was exclusively included in our study, it is uncertain whether these findings can be applied to other ethnic groups or research institute with different patient characteristics and procedural strategies [19]. Third, the follow-up time was still short, and there was no difference in in-hospital mortality, however, the drugs used in the past really improved the condition of myocardial infarction, which could not negate the long-term effect of the AAβ. Long term follow-up needs to be continued to illustrate the real-world results.

## Conclusions

In summary, the use of AAβ prior to myocardial infarction did not improve the in-hospital MACE; this may be the result of a short follow-up. However, AAβ did improve the cardiac function and reduced the infarct size. With the increase in follow-up time, we firmly believe that there must be showing the more benefits of medication and ultimately improving the MACE, These results should be confirmed by future dedicated large, randomized clinical trials with a long term follow-up.

## Abbreviations

ACEI: angiotensin-converting-enzyme inhibitors
ARB: angiotensin-receptor blockers
β-B: β-blockers
AAβ: ACEI/ARB/β-B
AMI: acute myocardial infarction
CHD: coronary heart disease
Myo: myoglobin
CK-MB: creatine kinase-myocardial band
cTnI: cardiac troponin I
EF: ejection fraction
FS: fraction shortening
MACE: major adverse cardiovascular events
CBD Bank: Cardiovascular Center Beijing Friendship Hospital Database Bank
STEMI: ST-segment elevation AMI
NSTEMI: non-ST-segment elevation AMI
BMI: body mass index
PCI: percutaneous coronary intervention
